# The Different Properties of Geopolymer Composites Reinforced with Flax Fibers and Carbon Fibers

**DOI:** 10.3390/ma17112633

**Published:** 2024-05-29

**Authors:** Francesca Brugaletta, Anton Frederik Becher, Danilo Laurent Rostagno, JeongHye Kim, José Ignacio Fresneda Medina, Celina Ziejewska, Joanna Marczyk, Kinga Korniejenko

**Affiliations:** 1 Faculty of Chemical Engineering for Industrial Sustainability, Department of Civil Engineering and Architecture, Università degli Studi di Catania (University of Catania), Piazza Università, 2, 95124 Catania, Italy, danilo.rostagno@gmail.com (D.L.R.); 2 Institut für Maschinenkunde und Fertigungstechnik, Technische Universität Bergakademie Freiberg, Gustav-Zeuner-Straße 7, 09599 Freiberg, Germany; antibe02@gmail.com; 3 Department of Bio-Fibers and Materials Science, College of Agriculture & Life Sciences, Kyungpook National University, 80 Daehak-ro, Buk-gu, Daegu 41566, Republic of Korea; gloria1211@knu.ac.kr; 4 Faculty of Mechanical Engineering, Polytechnic University of Valencia, Plaza Ferrándiz y Carbonell, 03801 Valencia, Spain; ignacio.fresneda.medina@gmail.com; 5 Faculty of Materials Engineering and Physics, Cracow University of Technology, 37 Jana Pawła II Street, 31864 Cracow, Poland; celina.ziejewska@pk.edu.pl (C.Z.); joanna.marczyk@pk.edu.pl (J.M.)

**Keywords:** geopolymer, carbon fiber, flax fiber, composite

## Abstract

The main motivation for this research was to improve the properties of geopolymers by reinforcement using synthetic and natural fibers, and to gain new knowledge regarding how the nature and/or the quantity of reinforcement fibers influences the properties of the final geopolymers. The main objective was to investigate the effects of different types of reinforcement fibers on the properties of the geopolymers. These reinforcement fibers were mainly environmentally friendly materials that can be used as alternatives to ordinary Portland cement. The authors used fly ash and river sand as the raw materials for the matrix, and added carbon fibers (CF), flax fibers (FF), or a hybrid of both (CFM) as reinforcements. The samples were prepared by mixing, casting, and curing, and then subjected to various tests. The main research methods used were compressive strength (CS), flexural strength (FS), water absorption (WA), abrasion resistance (Böhme’s disk method), microstructure analysis (SEM), chemical composition (XRF), and crystal structure analysis (XRD). The results showed that the addition of fibers partially improved the mechanical properties of the geopolymers, as well as reducing microcracks. The CF-reinforced geopolymers exhibited the highest compressive strength, while the FF-reinforced geopolymers showed the lowest water absorption. The authors, based on previous research, also discussed the factors that influence fiber-matrix adhesion, and the optimal fiber content for geopolymers.

## 1. Introduction

Geopolymers are becoming increasingly popular in the world market, both because of their sought-after environmentally friendly nature and their potentiality. The interest in this research arose from surprising statistical data, which reported a reduction of 80% in CO_2_ emissions during the manufacture of geopolymer composites compared to that of ordinary Portland cement [1]. Additionally, there are a lot of possible raw materials that can be used for their production, including industrial by-products such as fly ash. This is abundant worldwide and it is considered a good geopolymer binder based on its composition, making it a perfect waste to reuse [2]. In Glukhovsky’s Soil Silicates (1952), the importance of the use of local and natural raw materials was pointed out. One example could be the use of river sand in geopolymers, which strives to further reduce their cost and impact on the environment [3].

To optimize geopolymers for use in various fields, and to compensate for the brittle character of these materials, it is necessary to add reinforcing materials, and particularly intensive research has been carried out in recent years on fiber reinforcements. Natural fibers, such as bamboo, flax, hemp, and jute, are eco-friendly materials that can be used as reinforcements within a geopolymer matrix. These kinds of reinforcements provide benefits, such as improved tensile and flexural strength, reduced density, and improved thermal and acoustic insulation [4,5]. Despite their many advantages, natural fibers also present disadvantages, such as a heterogeneous structure and high water absorption [6,7]. It is a challenge to apply these fiber within composites, especially those intended for building purposes. An additional challenge in the synthesis of geopolymers relates to the degradation of natural components in alkaline media [6,7]. Because of this, it is necessary to carefully plan the properties of alkali solutions used for the preparation of these materials.

One of the main defects of geopolymers, which limits their current application, is that under adverse environmental conditions, these materials are prone to the formation of microcracks [8,9]. These microcracks may eventually coalesce, forming one or more macroscopic cracks, which can propagate and lead to structural damage. Due to this, carbon fiber (CF)-reinforced geopolymers are attracting increasing scrutiny from researchers, who have noted the ability of CFs to control macroscopic crack propagation through the bridging effect, delaying the initiation and expansion of microcracks and improving splitting tensile strength. Hence, a comprehensive investigation of CF-reinforced geopolymers is imperative for advancing the field [8,9].

Another focus of this research is the blending of natural and synthetic fibers to create so-called hybridized geopolymers. Scientists expect the hybrid properties of such fiber combinations to improve geopolymers, not only in comparison to unreinforced composites, but also in comparison to mono-fiber-reinforced composites. Rajendran et al. [10]’s evaluation of experiments conducted in this field showed that the addition of hybrid fiber blends changed the properties of geopolymers in almost every area. In particular, in the area of mechanical properties, the tensile strength, ductility, and impact strength were significantly improved. The hybridization of geopolymers is therefore proving to be a promising field of research [9,10]. It has been shown that unreinforced geopolymers have a brittle structure, which leads to their use in relatively few applications [11,12].

The main aim of this article is to research and study how the nature and/or the quantity of the reinforcement fibers used in geopolymers influences the properties of the final structures. This represents a novel research pursuit, as there have been only a limited number of previous works performed on hybrid reinforcement in geopolymers, especially in relation to joining natural fibers, such as flax fibers, with synthetic fibers, such as carbon fibers. For this study, carbon and flax fibers were selected because this combination has not been investigated previously in the context of geopolymer matrices. 

## 2. Materials and Methods

### 2.1. Materials

The fly ash (FA) used was sourced from the coal-fired power plant ‘Skawina’ (located in: Skawina, Lesser Poland, Poland). The FA was obtained by electrostatic precipitation of fine particles present in the exhaust gases of coal-fired furnaces [13]. The chemical composition of FA is presented in Table 1 and Table 2, showing that the main components of the sourced FA were silica and alumina. The high content ratio of these elements was favorable from the point of view of the geopolymerization reaction. 

**Figure 1:**
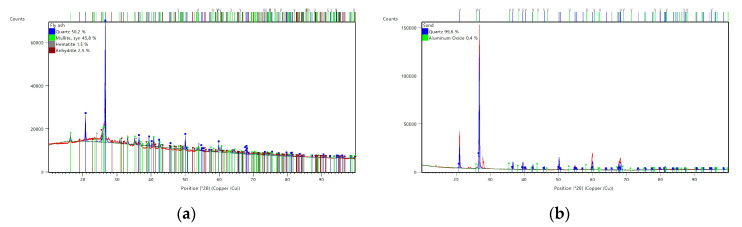
XRD diffractograms of raw materials for the geopolymerization process: (**a**) Fly ash from “CHP Skawina”, and (**b**) river sand (fine aggregate).

The mineralogical composition of the FA was determined, as seen in Figure 1a. The main components were quartz and mullite. This kind of composition is typical for FA used for the geopolymerization process [8,14].

**Figure 2:**
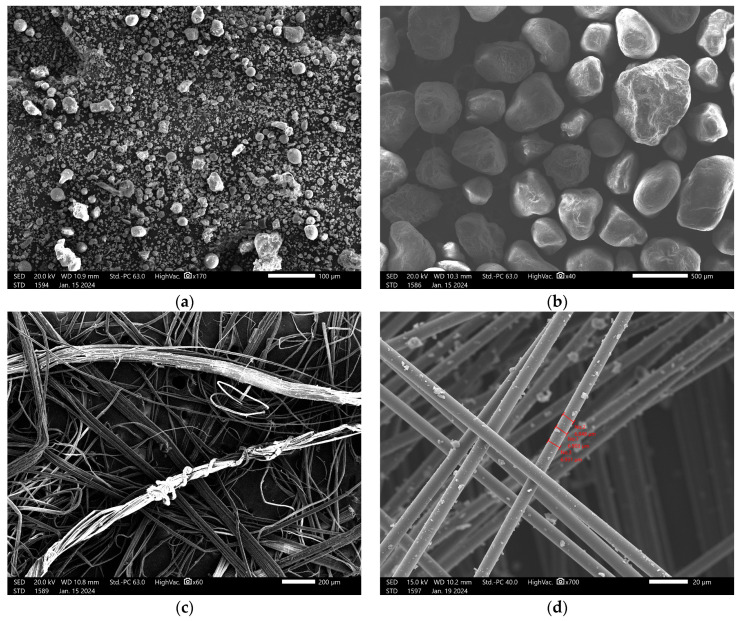
SEM micrographs: (**a**) Fly ash, (**b**) river sand, (**c**) flax fiber, and (**d**) carbon fiber, with diameter measurements.

The morphology of FA is presented in Figure 2a. FA includes a large amount of regular and spherical particles. This uniform shape improves the workability of the material, and during the preparation of the geopolymers, reduces the need for liquid substances [2]. It is beneficial from the point of view of creating composites with fibers that usually require a large amount of solution. 

A fine aggregate river sand (IVERSO, Iwiny, Poland) was also used. This type of sand comprises a mixture of rock particles, and obtains its shape through the chemical and physical abrasion of rock and stone formations [15]. These formation process and the presence of different rock types and environmental influences make this type of sand a material that presents strong regional differences. These affect, among other things, the sand composition. The high proportion of quartz (SiO_2_) in the earth’s crust, as well as its high hardness and abrasion resistance, make it the dominating part of the composition [15]. The chemical composition of the used sand is given in Table 1 and Table 2, and the mineralogical composition is shown in Figure 1b. According to the XRD analysis, the main mineral ingredient was quartz. The morphology is presented in Figure 2b. Due to its stable structure, the silicate in river sand also does not react with alkaline additives, making it a suitable component for geopolymers [3].

**Figure 3:**
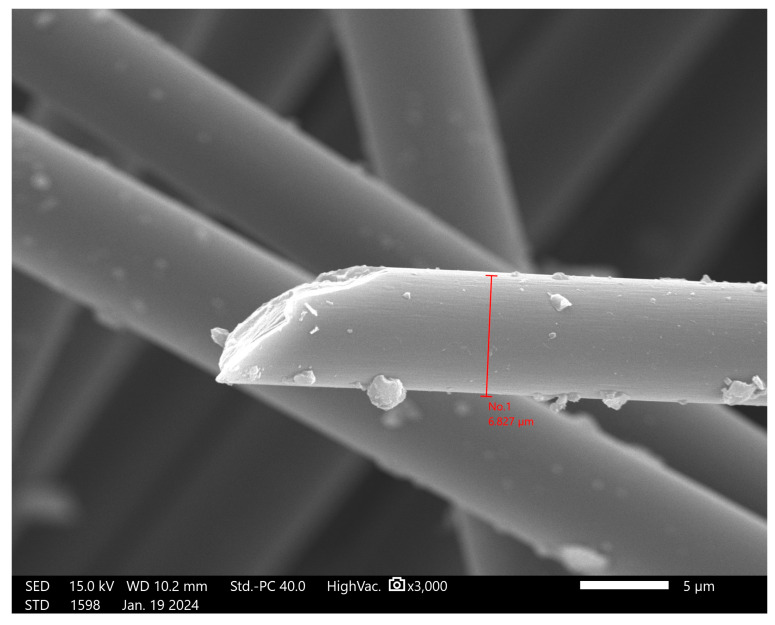
Measurement of the diameter of the carbon fiber.

Carbon fibers are synthetic fibers mostly composed of carbon atoms, and are about 5 to 10 μm in diameter. The atomic structure of carbon fiber is similar to that of graphite, a crystalline material consisting of sheets of carbon atoms arranged in a regular hexagonal pattern. Carbon fiber has become very popular in aerospace, civil engineering, military, motorsports, and other competition sport applications due to its properties, being used most notably as a reinforcement to obtain composite materials [16]. While carbon fibers may be woven into textiles to obtain sheets of material with a certain pattern and better mechanical properties, this study has only observed the effect of short fibers dispersed in the material with a random orientation. Short carbon fibers have advantages over other kinds of fibers because of their high modulus, high strength, low thermal expansion, and excellent electrical properties [16]. They have been observed to inhibit the spread of microcracks, enhancing material crack resistance. Carbon fibers are already being incorporated into cement-based materials to enhance tensile and bending strength, facilitate structural repair, and bolster the seismic and fatigue resistance of structures [8,17]. Additionally, carbon fibers possess advantageous durability properties, such as resistance to high temperatures and corrosion [8,17]. The carbon fibers (R&G GmbH, Waldenbuch, Germany) used for the samples were 3 mm in length, and the diameter was measured with SEM. The diameter was 6.827 μm, as shown in Figure 3. The artificial carbon fibers (Figure 2d) were perfectly cylindrical with smooth surfaces.

**Figure 4:**
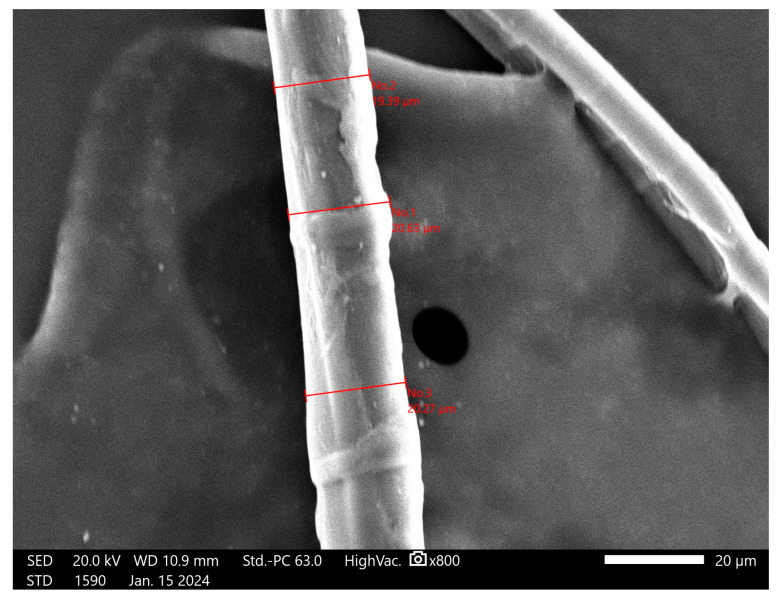
Measurement of the diameter of the flax fibers.

Natural fibers have significant advantages, including being eco-friendly, biodegradable, low-cost, and non-toxic. Furthermore, they have lower density and higher specific strength when compared with synthetic fibers [11,18]. According to chemical composition analyses, they are mainly made up of cellulose (70–85%) and hemicelluloses (11–20%), and other ingredients include pectin (2–12%) and lignin (approximately 2%) [7]. Since the short random fiber reinforcement of geopolymer matrices does not require a complex production process, their use is a field of study for some large-scale applications (e.g., building materials) [19]. Flax fibers, also wrongly known as linen, can be identified by their typical nodes, which provide flexibility and texture. The main difference between flax and linen is that the former represents the fibers from the flax plant, while linen is made of flax fibers, which are transformed into the textured final product after processing. The cross-section of the fibers is made up of irregular polygonal shapes, which are responsible for their rough texture. Flax fibers are mainly composed of cellulose, hemicellulose, and lignin, and these improve the biodegradability and recyclability of geopolymers. This natural fiber is widely used because it can absorb and lose water rapidly [19]. Flax fiber is preferred to cotton fiber for its strength and durability, even though it has low interfacial strength in composites and less elasticity. The flax fibres used in this research were sourced from the research organization ‘Instytut Włókien Naturalnych i Roślin Zielarskich Państwowy Instytut Badawczy’ (Poznań, Poland). Flax fibers (Figure 2c) are compact, and they do not split themselves during the mixing process; thus, they were split manually. The fibers were approximately 3 mm in length (after cutting) and presented various diameters (Figure 4). The major advantage of flax fibers is that their surfaces are rough, and this characteristic can promote coherency between the fibers and the matrix. The dimensions of the fibers that were added as reinforcement in this research were investigated using a scanning electron microscope. Considering the irregularity of the natural fibers, to evaluate the diameter of the flax fibers, three measurements were taken, as shown in Figure 4. The diameter of the flax fibers was considered as the average of these measurements, which was 20.10 μm.

To initiate the mixing process, the alkali solution was prepared a minimum of 2 h before the process. The solution was a 10 M mixture, composed of sodium hydroxide (NaOH), sodium silicate (water glass), and tap water. The ratio of sodium base to water glass was 1:2.5. The ingredients were applied in the following amounts: 314 g of NaOH, 686 mL of tap water, and 1700 g of water glass. 

Four types of samples were prepared: reference (pure geopolymer), a geopolymer with about 1% weight made up of CF, a geopolymer with about 1% weight made up of FF, and a hybrid geopolymer made up of about 0.5% weight in CF and about 0.5% in FF. The sample designations are presented in Table 3. This proportion of the fibers was applied because of the effect of varying the content of carbon and flax fibers on the workability properties of the samples. The workability of the paste increased with fiber addition, but above 1% weight, casting became difficult. To eliminate this negative influence, more solution was added to obtain similar workability to the reference samples. Both fibers were added without any pretreatment. Based on previous research, the pretreatment of flax fibers does not significantly change their properties in geopolymer matrices based on fly ash [20].

In this study, all of the samples used the same type of fly ash and river sand in a 50:50 proportion. The mix of fly ash and river sand was added to the mixing container. Subsequently, the solution was slowly poured and mixed for 5 min. In the case of the reinforced samples, adding the fibers by dividing them manually into small parts was necessary to avoid agglomeration and to make sure a homogeneous distribution was maintained. Each of the prepared samples was placed into a mold, which was used to obtain the shape specific for the required tests. Before curing to remove all of the air stacked inside the geopolymers, it was necessary to place the molds into a vibrator. After 2 min, the samples were ready to be cured. The samples were cured for 24 h at 75 °C in a laboratory oven. Next, they were de-molded and stored in the laboratory for 28 days. The samples then underwent tests to observe and analyze the influence of the reinforcements. 

### 2.2. Methods

Analysis of the compressive strength (CS) and flexural strength (FS) was performed to investigate how the carbon fibers and the natural fibers affected the mechanical properties of the geopolymers. In particular, the compressive and flexural strength tests were conducted on a 3000 kN compression testing machine (Matest, Treviolo, Italy). Before the test, two opposite sides of the sample were ground flat using sandpaper.

**Figure 5:**
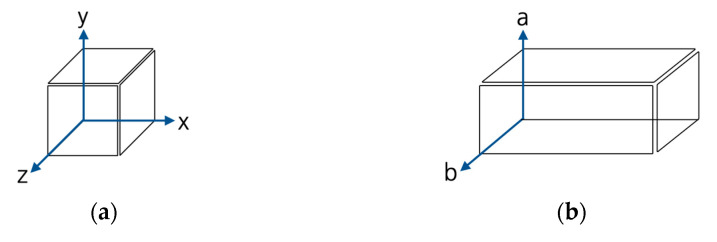
(**a**) Square for compressive strength test; (**b**) Rectangular for flexural strength test.

Compressive strength is the maximum stress that can be applied until the material is destroyed when subjected to a compressive force in one direction. In this study, the shape of the samples was cubic, and the measurement of the length was performed by taking into consideration the x, y, and z directions, as shown in Figure 5a. The samples were prepared in 50 mm × 50 mm × 50 mm molds.

The samples were positioned in the center on the lowest plate of the testing apparatus. The starting force was 0.500 kN, and the specimen was loaded with a continuous constant rate of 0.5 MPa/s. The found maximum load was the compressive strength of the sample. The compressive strength was calculated according to the following equation:

(1) $$CS=\frac{P}{A}\;$$

where *A* is the cross-sectional area, and *P* is the load on the sample.

Flexural strength is the material’s ability to resist deformation under load. In other words, flexural strength is a mechanical property that evaluates the stiffness of a material. For this test, the shape of the samples was a rectangular prism (cuboid), and the measurement of the length was performed by taking into consideration the a and b directions, as shown in Figure 5b. The samples were prepared in 40 mm × 40 mm × 160 mm molds. In this study, the flexural strength was measured using the three-point bending flexural test method. The specimen was placed on two supports (distance between supports 150 mm), and a load F was applied in the center of it. Failure occurred when the strain or elongation exceeded the limit of the material. The flexural strength, σ*_fs_*, was calculated according to the following equations:

(2) $$\sigma =\mathrm{stress}=\frac{M\cdot c}{l}$$

(3) $$\sigma_{fs}=\frac{3\cdot F\cdot L}{2\cdot b\cdot d^{2}}$$

where *M* is the maximum bending moment [N∙m], *c* is the distance from the center of the specimen to the outer surface [m], *I* is the moment of inertia of the cross-section [kg∙m^2^], F is the applied load [N], L is the distance between the support points [m], *b* is the width of the sample [m], and *d* is the highness of the sample [m].

The water absorption test is a fundamental test used to evaluate the water absorption properties of various materials, including cement. The main purpose of this test is to measure the amount of water that a given volume of material is able to absorb. This provides information about the porosity of the material and its ability to resist the adverse effects of water, such as freeze expansion and chemical degradation. The water absorption test procedure for geopolymers in this study involved the following steps:

1. Specimen preparation: Representative samples were selected and prepared in the form of cylinders or cubes, according to specific regulations.
2. Initial drying: Samples were carefully dried to remove any pre-existing moisture.
3. Immersion in water: The dried samples were completely immersed in water for a specified period. In the analyzed case, the material was immersed in water for 5 days, and the measurements were taken two times per day to understand the absorption capacity over time.
4. Subsequent drying: After immersion, the samples were removed from the water and dried again to remove excess surface water. This process was carried out each time the samples were taken out to take measurements.
5. Weight after absorption: The weight of the samples after water absorption was measured.

Standard laboratory instruments such as precision balances, immersion vessels and an oven were used to carry out the water absorption test. Water absorption was calculated as the difference in weight before and after immersion in water, expressed as a percentage of the initial weight. A higher percentage of absorption may indicate a higher porosity of cement, which could negatively affect its strength and durability.

Abrasion refers to the wearing down of surfaces due to abrasive grains. Abrasion resistance refers to the ability of materials to withstand wear. Abrasion resistance is used to determine the resistance of building materials produced for flooring, cladding, and pavements, and to demonstrate suitability for higher movement areas [21]. The abrasion resistance of the sample in the present study was measured using a Böhme disk (Matest, Treviolo, Italy). This testing apparatus consisted of a cast iron horizontal disc with a speed of 30 rpm and a diameter of 750 mm furnished with a 200 mm test track to position a specimen, a control panel with a digital revolutions counter capable of an automatic stop after preset revolutions, a specimen holder, and an adjustable charger used to produce a force of 294 N ± 3 N on the specimen. The test consisted of grinding the specimen’s surface during 22 revolutions of the Böhme disk, and calculating the wearing caused by abrasion after 16 cycles on each sample, turning the sample by 90° after each and every cycle, in order to produce even grinding in all directions. The sample used was a cube with sides of approximately 71 mm, with the contact surface free of irregularities and extraneous materials, and as flat as possible. The sample height was measured with a digital caliper, and its weight with a digital scale. As for the abrasive grains, approximately 20 g of artificial corundum, which has a hardness of 9.0 on the Mohs scale, was used per cycle. The measure of the wear was taken as the average decrease in the specimen’s volume. The measure of the specimen’s abrasion was the decrease in volume ΔV, calculated according to the following equation:

(4) $$\Delta V=\frac{\Delta m}{\rho r}$$

where ΔV is the decrease in the specimen’s volume after 16 cycles [mm^3^], Δm is the decrease in the specimen’s mass after 16 cycles [g], and ρr is the specimen’s density [g/mm^3^].

Observation of the microstructure of geopolymer composites gives information about the adhesion between the reinforcement fibers and the matrix. This can lead to a change in the mechanical properties. The microstructures of the samples were analyzed using a scanning electron microscope (SEM). The surface of the geopolymers needed to be electrically conductive, so a thin layer of gold was applied. This represents an excellent technique, as it makes it possible to reach very high magnifications. This research was conducted using a JSM-IT200 InTouchScope™ Scanning Electron Microscope (JEOL, Tokyo, Japan), and was performed based on the fibers, raw materials, and samples that were previously broken in the compressive test.

The chemical composition (elemental and oxide) of the specimens was determined using X-ray fluorescence spectroscopy (XRF). The research was conducted with the EDX-7200 from the company SHIMADZU (SHIMADZU EUROPA GmbH, Duisburg, Germany). The software used was PCEDX Navi (Version: EDX-7000P). The fundamental steps for the analysis were the sample preparation, and setting the parameters of the machine. In general, a sample for XRF analysis should have a perfectly flat surface, because irregular surfaces change the distance from the sample to the source and can introduce errors. A sufficient quantity of “loose” powder was put in the plastic support and covered by plastic foil. The requirement was to make sure that the surface of the plastic foil had no void spaces. Regarding the setting parameters, after placing the sample, a screen layout was shown. In this layout, it was necessary to set the analysis conditions, sample name, and the analysis that was to be conducted. The collimator diameter was changed to 5 mm to ensure that the sample surface was as homogeneous as possible, and the process was initiated.

The crystal structure analysis, i.e., analysis of the arrangement of atoms in a crystal, was performed through X-ray diffraction (XRD). This research was conducted with the AERIS from the company Malvern PANalytical (PANalytical, Almelo, The Netherlands). This is a quasi-automatic diffractometer, owing to the presence of a robot arm which automates the sample processing. A few steps were required to initialize the process: sample preparation, measurement programming, and interpretation of the results. By using the samples that were previously broken in the compressive strength testing, powders were produced. This process was performed via the use of a specific machine. These rough materials were placed in a centrifugal mill. In this research, the Ultra Centrifugal Mill ZM300 (Retsch Polska Verder Polska Sp. z o.o., Katowice, Poland) was used. This centrifugal mill reduced the size of the sample, and thanks to an integrated temperature monitoring system, kept the temperature constant to guarantee reproducibility for temperature-sensitive materials. The speed varied from 6000 to 23,000 rpm. Following this, the samples were ready to be placed in the XRD’s sample holders.

## 3. Results and Discussion

### 3.1. Compressive Test and Flexural Test of Geopolymers

**Figure 6:**
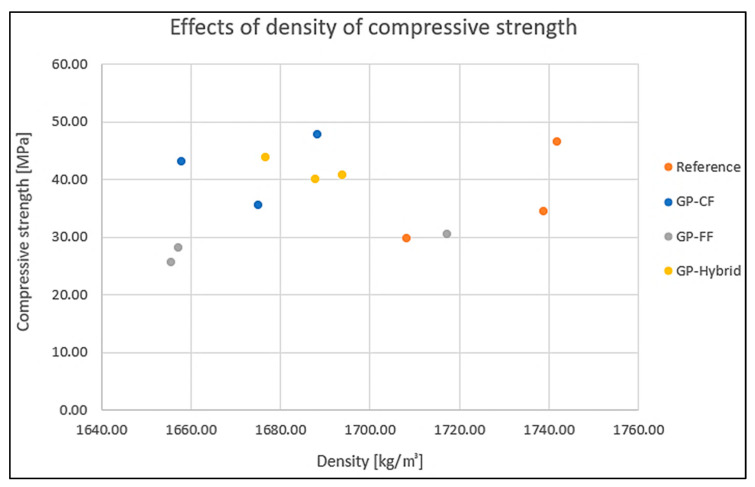
The effect of density on compressive strength.

In this paper, the compressive strength and flexural strength were tested for three samples per geopolymer composition. For the compressive test, the density of the specimens was calculated. In Figure 6 and Table 4, the results of the test are shown.

The density of the fiber-reinforced specimens was lower than that of the reference specimen. The correlation between the density and the compressive test was examined through a graph (Figure 6). The results of the study showed that there was no specific correlation between the density and the compressive strength. However, a correlation was noticed based on the calculation of basic values (Table 4). 

For each sample, the addition of fibers caused a reduction in the density of the composition. This is a predictable effect, which is connected with a lower density of the fibers compared to the geopolymer matrix [22]. Usually, the density should be correlated with the strength properties of the material [22,23]. However, in the case of fiber addition, this mechanism can be slightly different. The presented results show an increase in the compressive strength value, and at the same time, a decrease in density, in the case of usage of carbon fibers in the composition. This behavior is connected to the high strength of these fibers themselves [17]. The compressive strength was higher for the geopolymers reinforced with carbon fibers and with the mix of carbon fibers and flax fibers. On the other hand, when natural fibers were added to the geopolymer, the compressive strength radically decreased, which was in contrast to what is found in the literature. Other research has confirmed that the compressive strength should increase through reducing the porosity in the geopolymer [24].

**Figure 7:**
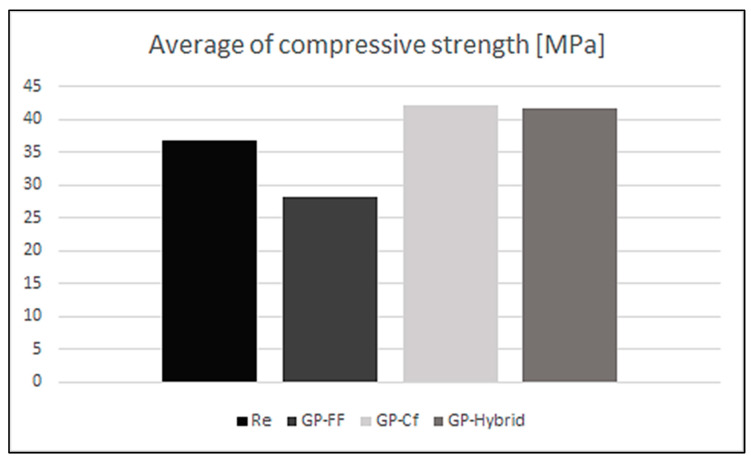
Average compressive strength.

The average values of compressive strength for the investigated compositions are presented in Figure 7. 

The best results for compressive strength were for the composition containing carbon fibers. The addition of flax fibers caused a reduction in compressive strength compared to pure matrix material. From this point of view, the addition of these fibers was ineffective. 

**Figure 8:**
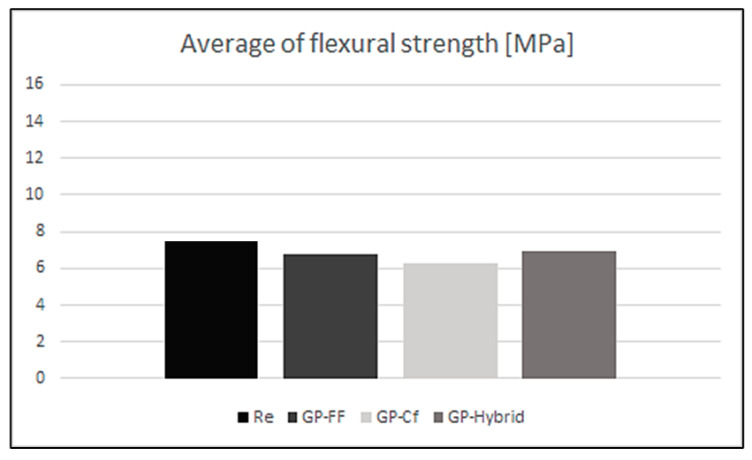
Average flexural strength.

The average values of compressive strength for the investigated compositions are presented in Figure 8. 

The best results were obtained for the pure matrix. The fiber addition, in both cases, did not significantly change the flexural properties of the materials. This was not typical behavior, because according to expectations based on the existing literature, fiber addition should improve flexural strength [22,25]. The literature suggests that the fiber cross-linking effect reduces brittleness by improving uniaxial tensile properties. Therefore, an improvement in the flexural strength of reinforced geopolymers was expected. However, this test showed different results; the reinforced samples instead showed decreased or similar strength values compared to the pure matrix [8]. In the flexural strength test, when fibers were added to the geopolymer, the flexural strength decreased. The cause of the decrease in flexural strength has been reported in other researchers’ results. For example, regarding CFs, studies have shown that a higher flexural strength value can be obtained with 0.5% fiber content than with 1% fiber content [26]. In the case of the presented research, the phenomenon of decreasing mechanical properties could be connected with the reduction in density, which could have been caused not only by fiber addition, but also by increasing the porosity and voids inside the materials. These kinds of defects are not visible on the surface, but they weaken the internal structure of samples, and thus impair their mechanical properties. This phenomenon may also have been related to the poorer workability of the mixtures containing fibers.

### 3.2. Water Absorption of Geopolymers

**Figure 9:**
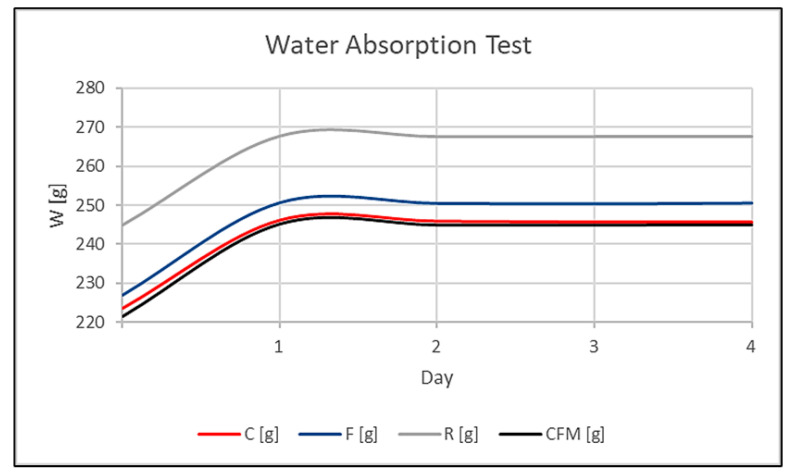
Water absorption test.

For this test, the behavior of the material submerged in water was observed. For five days, measurements were taken. The first measurement was taken on the dry sample with respect to the weight after leaving the piece submerged for 24 h. Before taking the measurements, the samples were left to dry for 10 min, and before placing them on the scales, the faces were dried using dry paper. The drying was performed in laboratory conditions (at a temperature of about 25 °C). After that, they were immersed again in the same container, and water was added if necessary. The results are presented in Figure 9 and Table 5.

As can be seen in Figure 9, the materials absorbed the maximum amount of water in the first 24 h, and no changes were observed during the following days.

The percentage of water absorbed in all samples was between 9 and 10%. This value was directly related to the porosity; as the manufacturing process was the same for all of the samples, the existence of fibers did not significantly change the porosity. However, some of the literature has shown that the addition of additives such as ceramic microspheres or fibers to geopolymers can improve their resistance to water absorption by decreasing pore connectivity and increasing matrix density [22,27]. According to other studies, specimens made by activation with higher alkali content have presented lower water absorption, apparent porosity, and water sorption [28].

### 3.3. Abrasion Test of Geopolymers

The abrasion test consisted of a comparison between the initial conditions and the final conditions. These conditions are shown in Table 6 and Table 7.

Looking at the results of the abrasion tests, it can be observed that the abrasive wear increased by 8.97% in the CF-reinforced GP, by 6.36% in the FF-reinforced GP, and by 27.91% in the GP reinforced with both fibers. This shows that the abrasion resistance of our GP decreased after the addition of fibers in its matrix, a detrimental result, and the opposite of what was expected to happen. Comparing these results to other previous research, it is shown that others have noted no significant improvements with the addition of carbon fibers [29], and that better results were found while using steel [30] and other fibres [31,32,33,34].

### 3.4. Microstructure of Geopolymers

**Figure 10:**
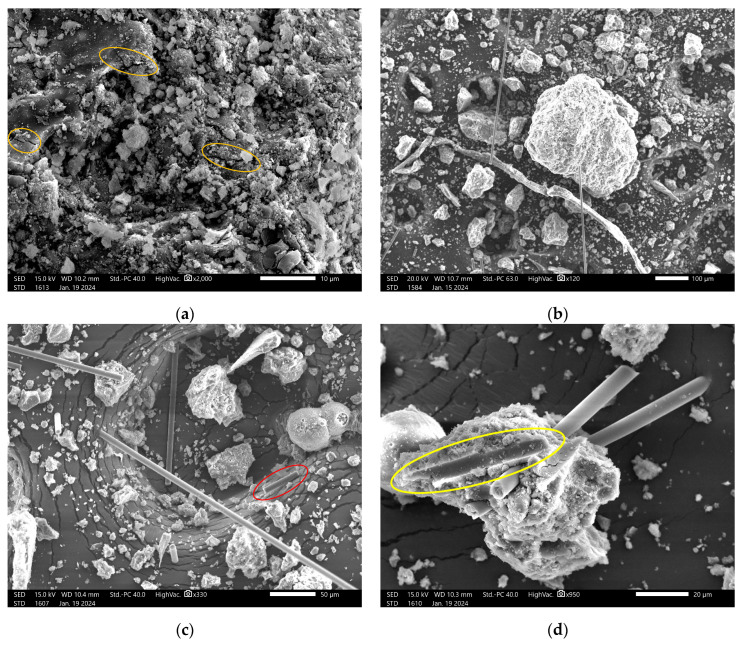

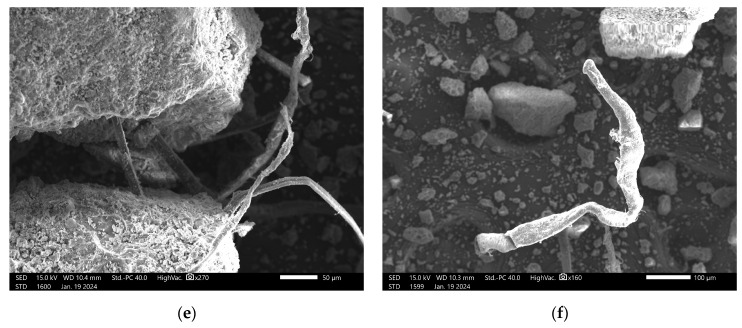
SEM micrographs of the geopolymers. (**a**) No reinforced geopolymers (reference sample); (**b**) geopolymers with 0.5% CF and 0.5% of FF; (**c**) CF geopolymers, with fiber fracture; (**d**) CF geopolymers, with fiber pulling out and cohesion with the matrix; (**e**) FF geopolymers, with fiber bridge; and (**f**) FF geopolymers, with deagglomerated fiber.

The microstructures of the samples were investigated using SEM. This technique also provided information about the adherence of the reinforcement fibers to the geopolymer matrix (Figure 10).

The fibers were randomly distributed. It was found that the more random the distribution, the more isotropic the behavior of the samples became. Figure 10a shows the reference sample (i.e., pure geopolymer). The circle highlights the presence of numerous microcracks within the geopolymer. Figure 10b shows the hybrid geopolymer, i.e., the geopolymer with 0.5% CF and 0.5% FF. It is possible to affirm that the fibers were randomly distributed, with a high amount of fractured carbon fibers. Figure 10c shows the CF geopolymers, and in particular, the red circle shows a fractured carbon fiber. Figure 10d provides a good explanation of the possible dual behavior of the fibers. 

The SEM investigation revealed that the pure geopolymers had multiple microcracks in the matrix, similar to results found in other research [8,27,35]. This phenomenon was not observed with the addition of the fibers, which can influence the mechanical properties of the material. This could explain the lowering of the mechanical properties in the case of fiber addition. The reinforced samples show what was expected. In particular, in Figure 10d, the yellow circle shows the pulling out of the fiber from the matrix, while the other two fibers had a good coherency with the matrix (at the end of the fibers, it is possible to see their fracture). It is confirmed that where there is a good bonding of the fiber and the matrix, the fiber tends to fracture; otherwise, if the bonding is weak, when stress is applied, the fiber pulls out [36]. Figure 10d also shows the undesired agglomeration of fibers. As shown in the literature, the clustering phenomenon increases when increasing the amount of fibers incorporated, and in particular when the content of CF is around 0.8–1% [6]. 

The flax composite showed a better coherency with the matrix, as shown in Figure 10e. The flax fiber performed the role of acting as the bridge to hold the matrix together. In contrast, Figure 10f shows a floating fiber, probably due to the pull-out or debonding phenomena. Increasing the content of the natural fibers to 1% led to better distribution and better mechanical properties [4].

### 3.5. Chemical Composition of Geopolymers

The elemental and oxide compositions of the geopolymer composites were investigated using XRF. As expected, the main elements of the geopolymers were silica (Si) and aluminum (Al). These elements were the main components of the raw materials (fly ash and river sand). The reinforcements did not change the elemental composition. The presence of sodium (Na), also confirmed using EDS analysis with SEM, was due to its use in the preparation of the specimens. Na was considered to be the “weight limit” element, so lighter elements, for example, carbon, were not able to be detected. Another element that was present in high amounts in the samples was iron (Fe), an abundant element in FA. The results are shown in Table 8.

The most abundant oxides were alumina, Al_2_O_3_, and silica, SiO_2_, typical for geopolymers. In addition, the samples also contained amounts of Fe_2_O_3_ and CaO. The results are shown in Table 9.

The XRF investigation confirmed what has already been discussed, and what is expressed in the literature [27,37]. Better still, the main elements were provided by the raw materials. This means that the quality of the fly ash and river sand used will affect the final quality of the specimens. An interesting finding was observed in the geopolymers containing mixed fibers: their composition, both elemental and oxide, was more or less the average of the geopolymers reinforced with only carbon or only flax fibers.

### 3.6. Crystal Structure of Geopolymers

**Figure 11:**
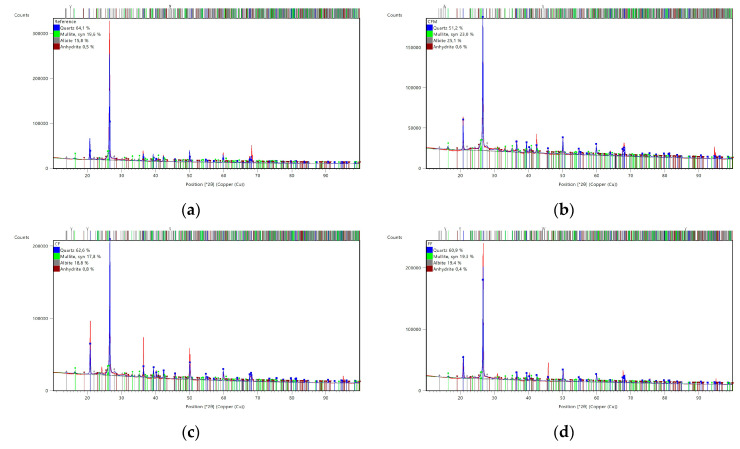
XRD analysis of the geopolymers. (**a**) No reinforced geopolymers (reference sample); (**b**) geopolymers with 0.5% CF and 0.5% of FF; (**c**) CF geopolymers; and (**d**) FF geopolymers.

The investigation of the mineralogical structure was performed using XRD analysis. All of the samples had similar (slightly different) crystal structures. The analysis results were corrected by manually examining reflection positions, peak intensities, and reflection shapes for all of the XRD patterns. The researched phases were quartz (SiO_2_), mullite (Al_6_Si_2_O_13_), hematite (Fe_2_O_3_), anhydrite (CaSO_4_), albite (NaAlSi_3_O_8_), and alumina (Al_2_O_3_). These phases are typical for a geopolymer matrix that is based on FA [14]. Quartz and mullite were present in the raw materials in larges. In particular, the amount of quartz was associated with the river sand. The results are shown in Figure 11.

The XRD analysis revealed similar peak patterns for the geopolymers (reinforced and pure) and for the fly ash. In particular, the primary component of the geopolymers was quartz, associated with the sand. There were no significant changes in intensity for the quartz or mullite peaks observed in the geopolymer samples, which can be explained by the fact that it is the amorphous phase of fly ash that undergoes geopolymerization at a high pH [38]. The presence of the albite can be associated with the strength enhancement region of the geopolymer matrix, and other research has shown that compressive strength increases by about 70 MPa with 40/50% addition of albite [38].

## 4. Further Directions for the Studies

Adding the reinforcement fibers did not result in a lot of changes. This can be explained by a lack of interaction between the fibers and the geopolymers during the polymerization process [39]. In the literature, similar results have been obtained for carbon-reinforced geopolymers [6,12]. There is a lack of literature relating to hybrid reinforcement; thus, the obtained results cannot be reinforced by any existing studies. 

Looking at the practical applications of the analyzed geopolymers, their mechanical properties, such as flexural and compressive strength, allow for applications in the construction industry [37,40]. Also, the results of the abrasion test were promising. According to the exemplary standard IS 1237 [41] “the wear shall not exceed 3.5 mm for general purpose flooring tile and shall not exceed 2 mm for heavy duty floor tiles” [42], and all of the examined samples respected these standards. All of the samples showed abrasive wear superior to 1.2 mm of height loss. The additional argument for this kind of application was the observed decreased water absorption.

This paper demonstrated the potential of using natural and synthetic fibers to enhance the properties of geopolymers for various applications, such as construction, insulation, and pavement, but further research is required. Prior to industrial applications, supplementary research should be performed, including aging tests, frozen-through investigations, and durability performance assessments, to confirm the materials’ properties over a long-term period.

## 5. Conclusions

The authors of this research investigated the properties of geopolymer composites reinforced with flax fibers and carbon fibers, as well as their combination. The authors conducted various tests to analyze the compressive strength, flexural strength, water absorption, thermal conductivity, abrasion resistance, microstructure, and chemical compositions of the samples. The most important findings were:

- The addition of carbon fibers showed an improvement in the compressive strength of the material, obtaining the highest value.
- Furthermore, it was observed that the addition of fibers did not improve the mechanical properties of the geopolymers in terms of flexural strength. As a result, the pure geopolymer showed the highest flexural strength.
- The authors also observed that the fibers reduced the water absorption and abrasion resistance of the geopolymers, indicating a higher porosity and lower durability of the composites.
- The SEM analysis revealed the microstructure and the fiber–matrix interface of the composites, showing different phenomena such as fiber fracture, pull-out, debonding, and bridging.
- The XRF and XRD analyses confirmed the chemical and crystal structures of the geopolymers and illustrated that they widely depend on the raw materials, showing the presence of alumina and silica as the main elements and oxides. Fiber addition did not significantly influence chemical composition.

## Figures and Tables

**Table 1:** The elemental composition of the raw materials, evaluated by XRF.

Element	FA, Content [%]	Sand, Content [%]
Si	45.830	94.258
Al	20.374	0.832
Fe	15.822	1.015
K	6.868	0.809
Ca	6.646	1.041
Ti	2.096	0.111
S	1.274	0.483
Mn	0.275	0.702
V	0.168	-
Sr	0.167	-
Zn	0.107	-
Zr	0.088	-
P	-	0.221
Cr	-	0.140
Yb	-	0.098
Au	-	0.075
Cl	-	-
Other elements	<0.05	<0.05

**Table 2:** The oxide composition of the raw materials, evaluated by XRF.

Compound	FA, Content [%]	Sand, Content [%]
SiO_2_	54.241	97.215
Al_2_O_3_	25.683	0.974
Fe_2_O_3_	8.894	0.376
CaO	3.997	0.399
K_2_O	3.802	0.270
TiO_2_	1.466	-
SO_3_	1.404	0.340
MnO	0.141	0.234
V_2_O_5_	0.111	-
SrO	0.067	-
Cr_2_O_3_	-	0.051
Other elements	<0.05	<0.05

**Table 3:** Composition of GP with NaOH solution, fly ash, sand, carbon fibers, and natural fibers.

Sample	Solution [mL]	FA [g]	Sand [g]	CF [g]	FF [g]
Reference	730	2000	2000	0	0
GP-CF	810	2000	2000	40	0
GP-FF	800	2000	2000	0	40
GP-Hybrid	825	2000	2000	20	20

**Table 4:** Compressive strength and density of samples.

Sample	Density [kg/m^3^]	CS [MPa]
Reference R_1_	1741.98	46.49
Reference R_2_	1738.95	34.42
Reference R_3_	1708.23	29.71
Average Reference	1729.72	36.87
GP-CF_1_	1675.00	35.60
GP-CF_2_	1688,31	47.80
GP-CF_3_	1657.94	43.12
Average GP-CF	1673.75	42.17
GP-FF_1_	1717.30	30.47
GP-FF_2_	1657.26	28.09
GP-FF_3_	1655.69	25.74
Average GP-FF	1676.75	28.10
GP-Hybrid_1_	1693.94	40.84
GP-Hybrid_2_	1687.74	40.02
GP-Hybrid_3_	1676.62	43.88
Average GP-Hybrid	1686.10	41.58

**Table 5:** Water absorption results.

Sample	Weight_0_ [g]	Weight_1_ [g]	Weight_2_ [g]	Weight_3_ [g]	Weight_4_ [g]	Absorption [%]
Reference (R)	244.96	267.76	267.62	267.63	267.65	9.25
GP-CF (C)	223.43	246.13	245.85	245.63	245.63	9.94
GP-FF (F)	226.95	250.71	250.54	250.40	250.62	10.33
GP-Hybrid (CFM)	221.38	245.23	244.96	244.95	245.01	10.65

**Table 6:** Initial conditions of the geopolymers.

Sample	Weight [g]	Height [mm]	Volume [cm^3^]	Density [g/ cm^3^]
Reference	666.38	71.95	363.699	1.837
CF	636.28	71.50	360.432	1.765
FF	642.68	70.96	357.709	1.797
CFM	636.82	71.87	362.297	1.758

**Table 7:** Final conditions of the geopolymers.

Sample	Weight [g]	Height [mm]	Weight Loss [g]	Height Loss [mm]	Volume [cm^3^]	Volume Loss [%]
Reference	650.77	70.62	15.61	1.33	8.498	2.343
CF	620.04	69.73	16.24	1.77	9.201	2.552
FF	626.67	69.33	16.01	1.63	8.909	2.491
CFM	617.73	70.07	19.09	1.80	10.859	2.997

**Table 8:** Element composition of the samples, evaluated by XRF for geopolymer composites.

Element	Reference Content [%]	GP-CF Content [%]	GP-FF Content [%]	GP-CFM Content [%]
Si	58.619	58.041	54.729	57.458
Al	13.758	13.833	14.122	13.417
Fe	12.503	12.485	14.303	13.020
K	5.828	6.201	6.333	6.294
Ca	5.732	5.875	6.400	6.145
Ti	1.603	1.657	1.806	1.723
S	0.880	0.825	0.992	0.804
Mn	0.287	0.284	0.308	0.287
V	0.136	0.137	0.152	0.156
Sr	0.101	0.118	0.136	0.126
Zn	0.084	0.084	0.106	0.090
Zr	-	0.065	0.077	0.068
P	-	-	-	-
Cr	0.281	0.227	0.168	0.227
Yb	-	-	-	-
Au	-	-	-	-
Cl	-	-	0.129	-
Other elements	<0.05	<0.05	<0.05	<0.05

**Table 9:** The oxide composition of the samples, evaluated by XRF for geopolymer composites.

Compounds	Reference Content [%]	GP-CF Content [%]	GP-FF Content [%]	GP-CFM Content [%]
SiO_2_	68.202	67.799	65.810	67.425
Al_2_O_3_	16.564	16.714	16.617	16.378
Fe_2_O_3_	6.492	6.552	7.598	6.903
CaO	3.217	3.328	3.752	3.502
K_2_O	3.005	3.172	3.266	3.272
TiO_2_	1.031	1.077	1.206	1.140
SO_3_	0.949	0.852	1.088	0.831
MnO	0.136	0.136	0.152	0.140
V_2_O_5_	0.093	0.096	0.109	0.111
SrO	-	-	0.051	0.050
Cr_2_O_3_	0.157	0.129	0.103	0.131
Other elements	<0.05	<0.05	<0.05	<0.05

## Data Availability

The original contributions presented in the study are included in the article, further inquiries can be directed to the corresponding author.
